# Sorafenib Inhibits Epithelial-Mesenchymal Transition through an Epigenetic-Based Mechanism in Human Lung Epithelial Cells

**DOI:** 10.1371/journal.pone.0064954

**Published:** 2013-05-31

**Authors:** Juyong Zhang, Yue-Lei Chen, Guanyu Ji, Weiying Fang, Zhaowei Gao, Yi Liu, Jun Wang, Xiaoyan Ding, Fei Gao

**Affiliations:** 1 Science & Technology Department, BGI-Shenzhen, Shenzhen, P. R. China; 2 The State Key Laboratory of Cell Biology, Institute of Biochemistry and Cell Biology, Shanghai Institutes for Biological Sciences, Chinese Academy of Sciences, Shanghai, P. R. China; 3 Department of Biology, University of Copenhagen, Copenhagen, Denmark; 4 King Abdulaziz University, Jeddah, Saudi Arabia; 5 The Novo Nordisk Foundation Center for Basic Metabolic Research, University of Copenhagen, Copenhagen, Denmark; Wayne State University, United States of America

## Abstract

The epithelial to mesenchymal transition (EMT) has been well recognized for many decades as an essential early step in the progression of primary tumors towards metastases. Widespread epigenetic reprogramming of DNA and histone modifications tightly regulates gene expression and cellular activity during carcinogenesis, and epigenetic therapy has been developed to design efficient strategies for cancer treatment. As the first oral agent approved for the clinical treatment of cancer, sorafenib has significant inhibitory effects on tumor growth and EMT. However, a detailed understanding of the underlying epigenetic mechanism remains elusive. In *this manuscript*, we performed a ChIP-seq assay to evaluate the activity of sorafenib on the genome-wide profiling of histone modifications. We demonstrate that sorafenib largely reverses the changes in histone modifications that occur during EMT in A549 alveolar epithelial cells. Sorafenib also significantly reduces the coordinated epigenetic switching of critical EMT-associated genes in accordance with their expression levels. Furthermore, we show that sorafenib potentiates histone acetylation by regulating the expression levels of histone-modifying enzymes. Collectively, these findings provide the first evidence that sorafenib inhibits the EMT process through an epigenetic mechanism, which holds enormous promise for identifying novel epigenetic candidate diagnostic markers and drug targets for the treatment of human malignancies.

## Introduction

As one of the most common types of human diseases, cancer is now the third leading cause of mortality in China and worldwide for both men and women, and more than 90% of deaths from cancer are due to the metastatic spread of primary tumors [1]. The breakdown of epithelium homeostasis, which leads to aggressive cancer progression, corresponds with the loss of epithelial properties and the acquisition of migratory mesenchymal characteristics, termed the epithelial-mesenchymal transition (EMT). This transition is believed to be an essential step for carcinoma invasion and metastasis [2], [3]. In addition to the essential role that this morphological transition plays in carcinogenesis, EMT has also been widely recognized in the context of physiological and pathological events such as embryonic development, wound healing and organ fibrosis. Interestingly, during EMT process, the loss of epithelial markers such as E-cadherin can occur through genetic or epigenetic mechanisms including aberrant DNA hypermethylation within the E-cadherin promoter [4]. This finding suggests that a program of molecular alterations leading to EMT, invasion and metastasis can be modulated epigenetically.

Epigenetic modifications, especially DNA methylation and histone modifications, work in concert with genetic mechanisms to regulate transcriptional activity in normal tissues. These modifications emerge as a dynamic and efficacious mechanism for the establishment and regulation of gene expression patterns during development, differentiation and tumorigenesis [5], [6]. Over the last decade, the epigenetic contribution of certain genes that are critical to EMT has been well studied. Notably, accumulating evidence has indicated that genome-wide reprogramming of histone modifications contributes to carcinogenesis and other EMT-related diseases, while bulk chromatin is globally reprogrammed during a pathological EMT [7], [8]. Because epigenetics has been found to be increasingly important in shaping the phenotypic variation of a broad range of biological events, further studies focusing on the mechanisms for epigenetic inheritance that underlie physiological and pathological EMT are highly desirable. These investigations may help us to identify epigenetically misregulated genes that can serve as diagnostic markers for epigenetic therapeutic strategies for treating cancer.

Recently, the successful development of small chemicals that disrupt several fundamental signaling pathways has signified a paradigm shift in medical therapies [9]. In addition to this established molecular-targeting strategy, insights into epigenetic profiles that identify molecular mediators of cancer drug sensitivity are leading to novel, promising means of prioritizing anti-cancer therapies. As a small molecular inhibitor that targets a number of serine/threonine and receptor tyrosine kinases, sorafenib (Nexavar or BAY 43-9006) is the first oral agent approved for the clinical treatment of a variety of tumor types [10], [11]. Previously, we uncovered a novel capacity of sorafenib to antagonize EMT and cell migration [12]. However, a detailed understanding of the underlying epigenetic mechanism is far from complete. In the current study, we performed chromatin immunoprecipitation followed by high throughput sequencing (ChIP-seq) to evaluate the effects of sorafenib on the genome-wide profiling of four selected histone modifications, and presented evidence that sorafenib inhibited TGF-β1-induced EMT through the epigenetic modulation of multiple EMT-associated genes in human alveolar epithelial cells.

## Results

### Sorafenib Suppresses TGF-β1-induced EMT in A549 Alveolar Epithelial Cells

**Figure 1:**
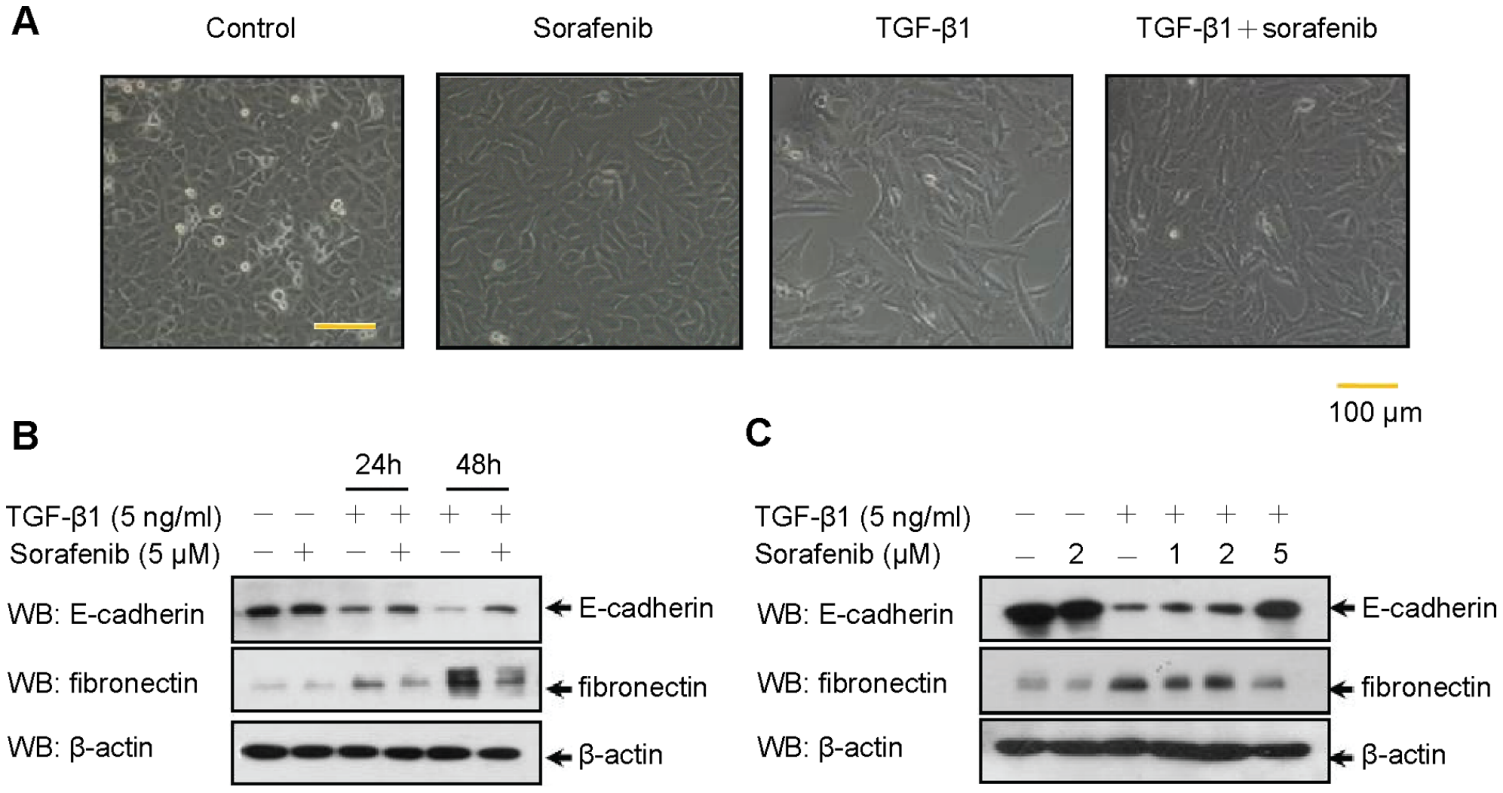
Treatment with sorafenib counteracts TGF-β1-induced EMT in A549 alveolar epithelial cells. (A) A549 cells were treated with TGF-β1 (5 ng/mL) and/or sorafenib (5 µM) for 48 h. Cells treated with vehicle only (DMSO) served as controls. EMT was determined based on morphological changes using a conventional phase-contrast microscope. The scale bar represents 100 µm for each section. (B) A549 cells were treated with TGF-β1 (5 ng/mL) and sorafenib (5 µM), lysed at different time points (0, 24 and 48 h) and immunoblotted using antibodies as indicated. (C) A549 cells were incubated with TGF-β1 (5 ng/mL) and increasing concentrations of sorafenib (1, 2 and 5 µM) for 48 h. The inhibitory effect of sorafenib on EMT was then assessed by Western blotting to detect the expression levels of related markers, including E-cadherin and fibronectin.

During metastasis, an increasing number of key growth factors have been found to be responsible for driving EMT, of which transforming growth factor-β (TGF-β) is a master inducer [13]. To examine whether sorafenib could impair TGF-β-induced EMT in epithelial cells, we used human A549 epithelial cells, a lung adenocarcinoma cell line that has been extensively used and is an ideal *in vitro* model for assessing EMT, carcinogenesis and drug metabolism [14]. Alveolar epithelial A549 cells underwent EMT after being exposed to TGF-β1 for 48 h, during which the cells lost their epithelial honeycomb-like morphology and obtained a spindle-like shape (Figure 1A). Along with these morphological alterations, the expression level of the adherens junction protein E-cadherin was decreased, whereas the expression level of the intermediate filament protein fibronectin was up-regulated (Figure 1B). Impressively, the treatment of A549 cells with sorafenib mediated a cellular resistance to EMT, as shown by cellular phenotypic alterations (Figure 1A) and the expression profiles of EMT markers (Figure 1B). We also treated cells with increasing concentrations of sorafenib under TGF-β1 stimulation. As shown in Figure 1C, sorafenib reversed TGF-β1-induced EMT in a dose-dependent manner. In addition, TGF-β1 provoked a marked increase in the migratory capacity of A549 epithelial cells that could also be abolished by sorafenib (Figure S1). Collectively, these data indicate that sorafenib counteracts TGF-β1-induced EMT and cell migration in A549 adenocarcinoma epithelial cells and provide a possible explanation for its effects with regard to tumor control and reduced cancer metastasis.

### Sorafenib Largely Restores the Changes of Histone Modifications during EMT

The findings above prompted us to further explore the underlying epigenetic mechanisms of EMT regulation by sorafenib. Histone modifications, but not DNA methylation, had been previously reported to undergo widespread reprogramming during EMT mediated by TGF-β1 [8]. We therefore focused on investigating dynamic changes in histone modifications on a genome-wide scale. We chose conventional markers of active euchromatin such as H3K9ac and H3K4me3, and contrasted their architecture with the repressive structures associated with H3K27me3 and H3K9me3. The profiling of these four selected histone modifications was performed using ChIP-seq on control, TGF-β1-treated and TGF-β1+sorafenib-treated cells (as indicated in Figure 1A and Figure S1). The experiments produced approximately 15 to 26 million uniquely mapped reads per chromatin modification (Table S1). Visualization of the ChIP signals indicated a high enrichment of H3K9ac and H3K4me3 around the transcription start sites (TSSs) and a broad distribution of the H3K27me3 and H3K9me3 signals in genic regions (Figure S2A). Subsequently, we focused on the changes of histone modifications in the promoter regions, which are important regulatory elements for gene transcription. We began by looking at the overall signal distribution of histone modifications in the A549 cells under different treatment conditions. Interestingly, we observed a global decrease in H3K9ac and a prominent increase of H3K27me3 upon TGF-β1 stimulation. The loss of H3K9ac and the gain of H3K27me3 in TGF-β1-treated cells were largely reversed after treatment with sorafenib (Figure S2B). Moreover, compared to control epithelial cells, the H3K9ac level was particularly highly up-regulated in TGF-β1+sorafenib-treated cells, indicating that sorafenib has much broader function than is currently known as an effective inhibitor against TGF-β signaling. Nevertheless, it appears that to a certain extent sorafenib exerts considerable reciprocal effects on H3K9ac and H3K27me3 changes that are stimulated by TGF-β1 during EMT. On the other hand, such effects on the active H3K4me3 mark and the repressive H3K9me3 mark were not as obvious on the global scale.

**Figure 2:**
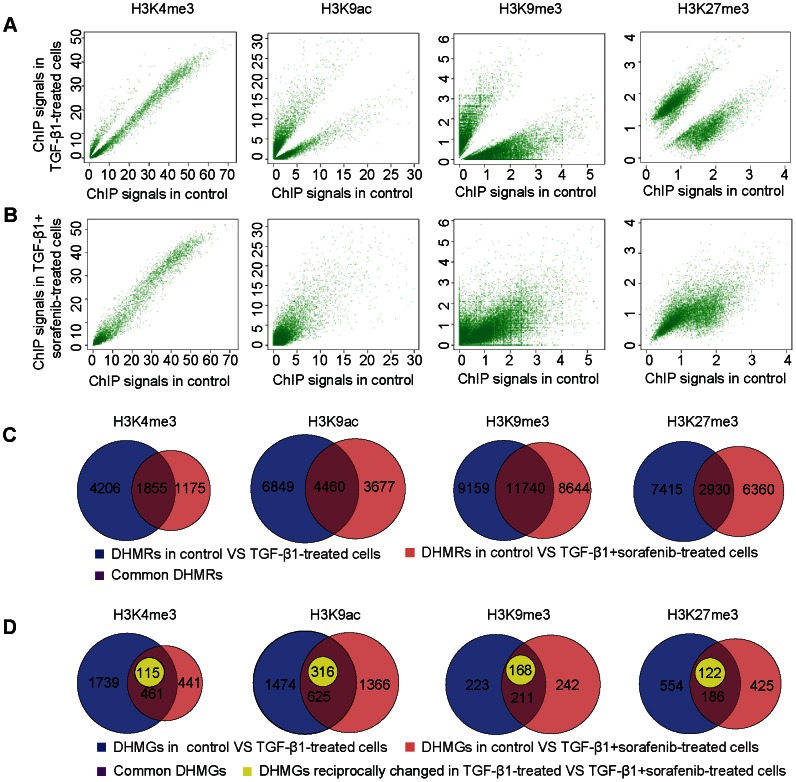
Sorafenib substantially reverses the histone modification changes associated with TGF-β1-induced EMT. We performed genome-wide analyses of four selected histone modifications in A549 cells under different treatment conditions as indicated in Figure 1A. (A, B) Scatter plots of the average ChIP signals of the significant DHMRs for each histone modification (H3K4me3, H3K9ac, H3K9me3 and H3K27me3) that were identified from comparisons between control and TGF-β1-treated cells. The X-axis indicates the average ChIP signal value of the DHMRs in control cells, while the Y-axis indicates the ChIP signal values of the same DHMRs in TGF-β1-treated cells (A) or in TGF-β1+sorafenib-treated cells (B). Each green spot indicates a DHMR. (C) Venn diagrams showing the relationship between DHMRs identified during EMT (blue), DHMRs identified between control and TGF-β1+sorafenib-treated cells (pink) and their intersection (dark magenta). (D) Venn diagrams showing the results of cross-matching DHMGs identified during EMT (blue) and DHMGs identified from a comparison between control and TGF-β1+sorafenib-treated cells (pink). The common DHMGs are indicated as the intersection (dark magenta), A portion of the genes shown in (dark magenta) contain reciprocal changes between DHMGS identified during EMT (blue) and DHMGs identified from a comparison between control and TGF-β1+sorafenib-treated cells (pink), and these are indicated with a yellow color.

We further performed pair-wise comparisons among the three treatment conditions to assess the changes in the histone modifications within specific genomic regions during EMT. Significant differential histone modification regions (DHMRs) were defined based on strict criteria (see Methods). By cross-matching the DHMRs identified between control and TGF-β1-treated cells with those between control and TGF-β1+sorafenib-treated cells, we found that 44%–72% of the DHMRs between control and TGF-β1-treated cells disappeared in the TGF-β1+sorafenib-treated cells (Figure 2C). Furthermore, the high divergence in the DHMR signals between control and TGF-β1-treated cells (Figure 2A) was largely restored by the treatment with sorafenib, as indicated by the scatter plots for the DHMR signals in corresponding regions between control and TGF-β1+sorafenib-treated cells (Figure 2B). We subsequently screened the genes that contained DHMRs in their promoter regions, which we term as differential histone modification genes (DHMGs). Cross-matching of the DHMGs revealed only a small proportion (21%–49%) of common DHMGs, among which the changes in histone modifications in a portion of the DHMGs between control and TGF-β1+sorafenib-treated cells were reciprocal to those between control and TGF-β1-treated cells (as indicated by the “yellow” cycle in Figure 2D). By combining the reciprocal DHMGs with the DHMGs that were specific to TGF-β1-treated cells, we obtained a collection of approximately 70%–91% of DHMGs that were reciprocally regulated by sorafenib for use in subsequent analysis.

### Sorafenib Significantly Suppresses Coordinated Epigenetic Switching of Critical EMT-associated Genes

Next, we used the set of DHMGs identified above to examine whether sorafenib exerts functional effects on the progress of EMT. To address this question, KEGG (Kyoto Encyclopedia of Genes and Genomes) pathway enrichment analysis was performed [15]. DHMGs and enriched pathways are summarized in Dataset S1. As expected, most of the top enriched pathways were involved in EMT-related functions, including cell adhesion, cytoskeleton remodeling, cell migration, MAPK signaling and EMT-related cancers, which is in good accordance with the results from DHMGs during EMT upon stimulation with TGF-β1 (Dataset S2). Notably, many of the DHMGs regulated by the two active marks (H3K4me3 and H3K9ac) were identified as enriched. A secondary measure of pathway enrichment analysis by DAVID (The Database for Annotation, Visualization and Integrated Discovery) revealed similar results (Dataset S3), indicating sorafenib exerts potent effects on the progress of EMT via epigenetic mechanism.

**Figure 3:**
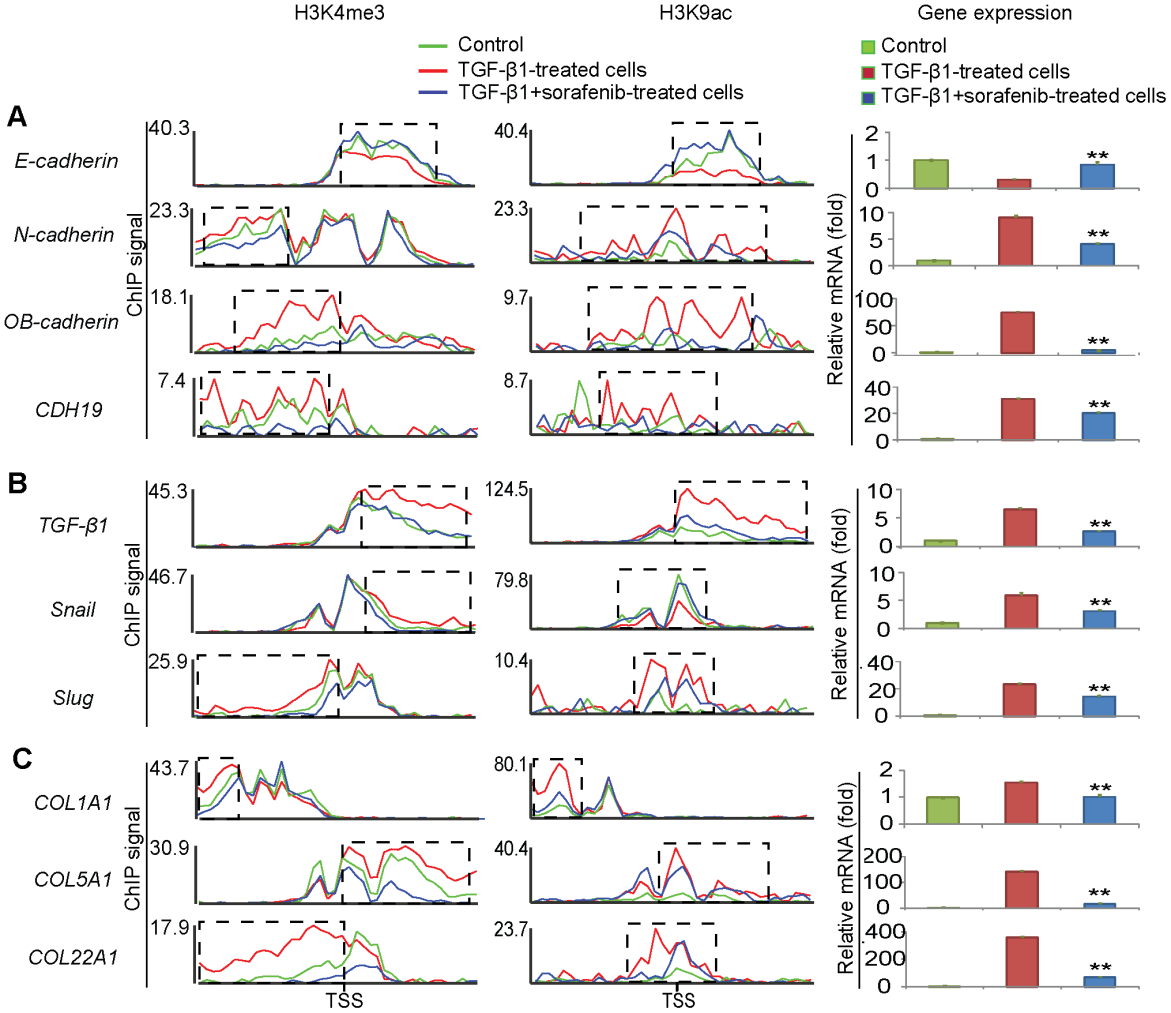
Sorafenib significantly suppresses coordinated epigenetic switches of critical EMT-associated genes. (A) Distribution of histone modification signals in the 4,000 bp promoter regions around the transcriptional start sites (TSS) (left) and gene expression (right) of *E-cadherin*, *N-cadherin*, *OB-cadherin* and *CDH19* in control (green), TGF-β1-treated (red) and TGF-β1+sorafenib-treated cells (blue). (B) Distribution of histone modification signals in the promoters (left) and gene expression (right) of *TGF-β1*, *Snail* and *Slug* in control, TGF-β1-treated and TGF-β1+sorafenib-treated cells. (C) Distribution of histone modification signals in the promoters (left) and gene expression (right) of *COL1A1*, *COL5A1* and *COL22A1* in control, TGF-β1-treated and TGF-β1+sorafenib-treated cells. Significant differential histone modification regions (DHMRs) induced during EMT and reciprocally regulated by sorafenib are marked by dotted frames. Gene expression data are representative of three similar experiments and displayed as the mean ± SE. **, *p*<0.01 as evaluated using the Student’s *t*-test.

In previous studies, many clusters of genes were found to play important roles during EMT progress. To gain further insight into the impact of sorafenib on EMT, we next focused on the epigenetic switching of three sets of genes that are critical for EMT. Among these EMT-associated genes, a switch from E-cadherin to N-cadherin expression is the most well characterized event [16]. In our results, the H3K4me3 and H3K9ac levels within the promoter region of *E-cadherin* were decreased and both these two active modifications in the promoter of *N-cadherin* were increased during EMT, which is consistent with the changes in their expression levels (Figure 3A). In addition, we found that another two adhesion molecules (OB-cadherin and CDH19) from the cadherin superfamily showed increased levels of H3K4me3 and H3K9ac in their promoter regions. Impressively, EMT induced these epigenetic switching and expression profiles of cadherins, and these changes could be significantly blocked by sorafenib (Figure 3A). The TGF-β signal triggers EMT primarily via a canonical Smad-dependent mechanism and induces the transcription of target genes responsible for EMT, including EMT-related transcription factors and extracellular matrix proteins (ECM) [17], [18]. Here, compared to cells undergoing EMT, sorafenib largely reduced the degree of H3K4me3 and H3K9ac marks within the promoters of *TGF-β1*, *Snail* and *Slug*, except H3K9ac of *Snail* (Figure 3B). As for *Snail,* the H3K9ac level within the promoter was decreased during EMT and this alteration was almost completely counteracted by sorafenib. Accumulation of ECM proteins promotes the progression of cancer by promoting cellular transformation and metastasis [19]. Based upon epigenetic profiling, we found that the promoter regions of several ECM genes associated with cell migration (for example, *COL1A1*, *COL5A1* and *COL22A1*) were dominated by H3K4me3 and H3K9ac following EMT and these switches were also significantly suppressed by sorafenib (Figure 3C). Additionally, we performed real-time qPCR to detect the consistency between epigenetic switching and gene expression profiling, and found that sorafenib could significantly suppress coordinated switching of critical EMT-associated genes at both epigenetic and expression level. Other than the selected genes above, the epigenetic modifications of other EMT-related genes were substantially regulated by sorafenib (Dataset S1), indicating that sorafenib functions as a potent inhibitor of EMT through the epigenetic modulation of multiple EMT-associated genes.

We further employed another lung carcinoma cell line (PC9 cells) to corroborate and extend the epigenetic mechanism of anti-EMT actions of sorafenib. Similar to the findings in A549 cells, PC9 cells treated with TGF-β1 also underwent EMT and this process could be counteracted by sorafenib (Figure S3A). We next used ChIP-qPCR analysis to evaluate the changes of H3K4me3 histone modification on the promoters of EMT-associated genes. As shown in Figure S3B, sorafenib significantly suppressed switching of many EMT-associated genes in PC9 cells (*p*<0.05, *t-*test), suggesting a general anti-EMT action of sorafenib.

### Sorafenib Potentiates Histone Acetylation by Regulating the Expression Levels of HATs and HDACs

**Figure 4:**
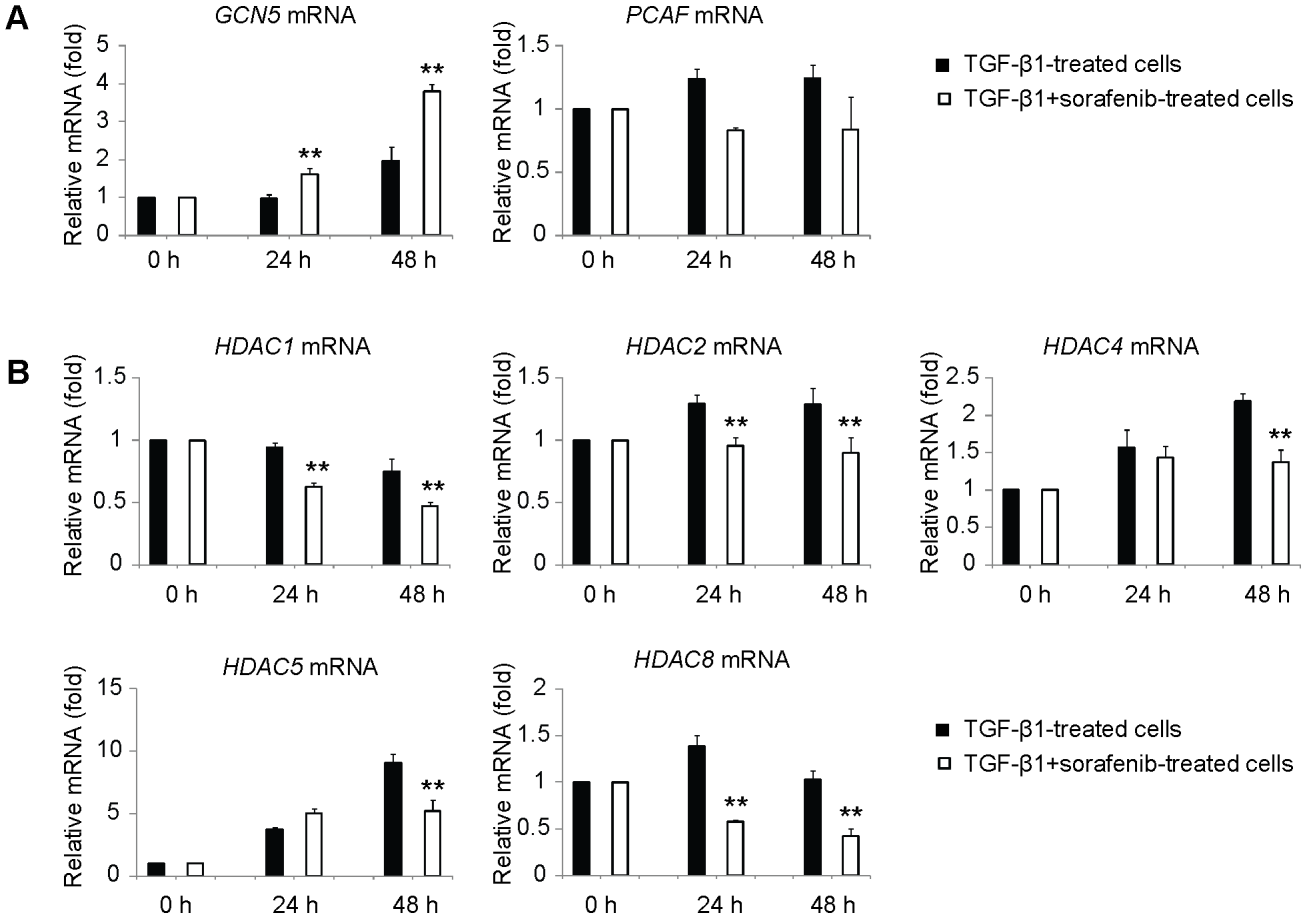
Sorafenib regulates the expression levels of HATs and HDACs. After treatment with TGF-β1 (5 ng/ml) in the absence or presence of sorafenib (5 µM) for 24 h or 48 h, A549 cells were subjected to real-time qPCR analysis to determine the effect of sorafenib on the expression levels of (A) HATs and (B) HDACs. Normal A549 cells without any treatment (at 0 h) were taken as the control. The quantitative ratios are shown as relative values that were normalized to GAPDH expression. The data are representative of six similar experiments and are displayed as the mean ± SE. **, *p*<0.01 as evaluated using the Student’s *t*-test.

The results presented above indicate that the inhibition of EMT by sorafenib was accompanied by an increase in the activating histone modification H3K9ac (Figure S2), which led us to further examine the impact of sorafenib on the expression of histone-modifying enzymes. H3K9ac is specifically mediated by GCN5 and PCAF, which are two highly homologous histone acetyltransferases (HATs) expressed in mammals [20], therefore, we hypothesized that sorafenib could potentially affect the expression levels of these two HATs. To test this hypothesis, we employed real-time qPCR to detect the changes in the expression of HATs during EMT, and found that the transcriptional level of *GCN5* was up-regulated in response to external TGF-β1 stimulation. Moreover, exposure of A549 epithelial cells to sorafenib eventually resulted in a significant increase in the expression of *GCN5* but had only a subtle effect on the mRNA level of *PCAF* (Figure 4A), suggesting that sorafenib increases the activating histone modification H3K9ac in A549 epithelial cells through the selective upregulation of *GCN5* transcription.

Because histone deacetylases (HDACs) also contribute to chromatin remodeling by catalyzing the removal of acetyl groups on the NH_2_-terminal lysine residues of the core nucleosomal histones [21], we subsequently evaluated the involvement of sorafenib in the modulation of HDAC expression. As shown in Figure 4B, the expression levels of the HDACs (*HDAC1*, *HDAC2*, *HDAC4*, *HDAC5* and *HDAC8*) were enhanced during EMT, whereas these changes were reciprocally blocked in TGF-β1+sorafenib-treated A549 cells, suggesting that sorafenib acts as a potential inhibitor of a large group of HDACs. Taken together, these results show that sorafenib potentiates the activating histone modification H3K9ac by regulating the expression levels of histone-modifying enzymes.

## Discussion

As the first oral agent in cancer therapy, sorafenib has been approved by the FDA for patients with several types of tumors since 2005 [10], [11]. Previous studies have heretofore focused on the ability of sorafenib to potently inhibit tumor growth and angiogenesis through blocking a number of receptor tyrosine kinases [10], [11], [22]. However, in addition to the established functions in clinical trials, sorafenib likely has much broader effects than is currently known. The present study sheds light on the novel capacity of sorafenib to reverse coordinated epigenetic switching at both the global level and the local level of critical EMT-associated genes that have been previously shown to play crucial roles during EMT, providing a possible epigenetic mechanism for its clinical benefits in patients with cancer metastasis, organ fibrosis and other EMT-related diseases. Using A549 carcinoma epithelial cells as an *in vitro* model, we found that treatment with sorafenib in during TGF-β1-induced EMT resulted in a loss of active histone markers (H3K4me3 and/or H3K9ac) at the promoters of *TGF-β1*, *Smad2/3* and downstream EMT mediators such as *Snail* and *Slug* (Figure 3). Combined with our previous finding that sorafenib disrupts the phosphorylation of Smad2/3 and STAT3 [12], we suggest that sorafenib interferes with TGF-β1-induced EMT via a dual mechanism–first via the direct inhibition of targeted kinase phosphorylation and then via transcriptional repression of EMT-related genes by epigenetic regulation. In addition to TGF-β signaling, we found that sorafenib inhibits the coordinated epigenetic switching of a broad range of signaling pathways that have been attributed to many oncogenic disorders, including Ras/Raf/MAPK and ErbB signal transduction (Dataset S1). Earlier studies on the resistance and sensitivity to sorafenib investigated gene-drug correlations by transcription profiling [23], [24], [25]. Here, we uncover a novel effect of sorafenib on the genome-wide profilings of epigenetic modifications in human lung adenocarcinoma cells, which holds enormous promise for the study of pharmacoepigenetics and interindividual variation in drug response and toxicity. A more detailed set of such investigations is therefore highly desirable to identify new epigenetic candidates that are sensitive to sorafenib and to ultimately develop personalized treatment of human malignancies.

Here, we provide the first blueprint of the dynamic pattern of epigenetic changes to EMT-associated genes during TGF-β1-induced EMT. The functional disturbance of cell-cell contacts that correlate with the loss of E-cadherin expression is a prerequisite for EMT, invasion and metastasis of tumor cells. The epigenetic silencing of E-cadherin has been well studied in many laboratories. For example, the evidence was first presented that the promoter hypermethylation was a possible mechanism of E-cadherin inactivation and later pointed to progressive histone modifications including H3K4me3 and H3K27me3 [26], [27], [28]. Our findings add to the short list of epigenetic silencing mechanisms of E-cadherin and show that a significant loss of H3K4me3 and H3K9ac accompanies the decrease in *E-cadherin* expression during EMT in lung epithelial cells. Impressively, we found that the expression of *OB-cadherin* and *CDH19*, two members of the cadherin superfamily, were clearly increased during EMT and were accompanied by an increase in both H3K4me3 and H3K9ac active marks at their promoters (Figure 3A). Many reports indicate that OB-cadherin contributes to EMT in pulmonary fibrosis and promotes the metastasis of several types of cancer cells [29], while very little information on the role of CDH19 is available. Due to the expression changes of CDH19 and the influence of sorafenib on the histone modification profiling at its promoter during EMT, we hypothesize that CDH19 could act as another vital mediator of EMT and a novel therapeutic target for the treatment of organ fibrosis and cancer. Additional investigation is certainly necessary, however.

In general, histone acetylation enables genes to be expressed, and HDAC1/2 activity is required to repress epithelial markers during cancer cell invasion and metastasis [30]. Recently, the inhibition of HDACs has become a focus of attention for the discovery and development of anticancer drugs because they have emerged as potential strategies for reversing the aberrant epigenetic changes that are associated with tumorigenesis [31], [32]. Till now, several classes of HDAC inhibitors have been approved to be successfully used as anti-cancer agents, some of which could be synergized with sorafenib in a combination therapy for diverse preclinical models of cancer [33], [34]. However, very little is known regarding the molecular mechanisms that underlie the synergistic interactions between sorafenib and HDAC inhibitors. In the current study, we interpret that these synergistic effects are at least partly because of a dual role of sorafenib in the increase of HAT expression and a decrease in HDAC expression (Figure 4). In addition to the impact of sorafenib on the expression of histone-modifying enzymes, we also observed that sorafenib attenuated the expression of DNA methyltransferases (DNMTs) (Figure S4). Based on these findings, the anticancer mechanism of sorafenib could be considerably broader and more complicated than originally thought to be a multikinase inhibitor.

To the best of our knowledge, this work presents the first report of sorafenib inhibiting TGF-β1-induced EMT through a possible epigenetic mechanism and shows that sorafenib largely reverses the coordinated histone modifications and subsequent epigenetic switching of relevant genes that are critical for EMT. As in other physiological contexts, epigenetic reprogramming during EMT is mediated by widespread changes in histone modifications. Further studies are needed to assess the consistency of these epigenetic changes between cell lines and in animal models, and to eventually understand the utility of epigenetically changed genes as diagnostic pipelines and drug targets for the treatment of human malignancies.

## Materials and Methods

### Reagents and Antibodies

Recombinant human TGF-β1 was purchased from *R&D* Systems (Minneapolis, MN, USA). Sorafenib (Nexavar, BAY 43-9006) was manufactured by Bayer Pharmaceuticals (West Haven, CT, USA). Primary antibodies against H3K9ac, H3K4me3, H3K27me3 and H3K9me3 were purchased from Millipore (Bedford, MA, USA). The rabbit monoclonal antibody against E-cadherin was purchased from Cell Signaling Technology (Beverly, MA, USA). Other primary antibodies described in this paper, including the anti-fibronectin and anti-β-actin antibodies, were purchased from Sigma-Aldrich (St. Louis, MO, USA).

### Cell Culture

Human alveolar epithelial A549 cells were obtained from ATCC (Manassas, VA, USA) and cultured in Dulbecco’s modified Eagle’s medium (DMEM) supplemented with 10% fetal bovine serum (FBS) (Biochrom, Berlin, Germany) at 37°C with 5% CO_2_.

### Western Blotting

To detect the protein levels of epithelial and mesenchymal markers, cells treated as indicated were lysed with 200 µl of lysis buffer and subjected to western blot analysis [35]. Approximately 50 µg of total protein was separated by SDS-PAGE, transferred to a PVDF membrane and incubated with the appropriate antibodies as indicated in the figure legends. The protein bands were visualized using an enhanced chemiluminescence (ECL) detection kit (Amersham).

### Chromatin Immunoprecipitation Sequencing

Chromatin immunoprecipitation (ChIP) was performed as previously described [36]. Briefly, cells were rinsed with room temperature PBS and cross-linked by 1% formaldehyde following sonication by a Bioruptor^TM200^ to generate chromatin fragments between 100 and 500 bp. The solubilized chromatin fragments were then immunoprecipitated with antibodies against H3K9ac, H3K4me3, H3K27me3 and H3K9me3, respectively. The ChIPed DNA was used for cluster generation and standard single-end sequencing with 50 bp reads (SE50) using Illumina Hiseq 2000.

### Processing of ChIP-sequencing Reads

After SE50 sequencing, the Illumina reads were post-processed and aligned to the human reference genome (UCSC hg19) using SOAP (Short Oligonucleotide Analysis Package) 2.01 with default parameters that excluded reads with more than five mismatched bases [37]. Reads that were mapped to more than one position in the genome were then filtered out. Multiple reads mapping to the same position were counted only once to remove potential bias from PCR. The uniquely mapped single-end reads were then extended to 150 bp to approximate the average size of the sequenced fragments, and these extended reads were used for subsequent analysis. We calculated the number of sequencing reads covering a specific base as the signal value for the histone modification for that base in the genome, and then we normalized the signal by equalizing the total data amount to 1 G bp for all samples.

### Defining the Differential Histone Modification Regions

Differential histone modification regions (DHMRs) for sample-to-sample comparisons were identified based on three strict criteria: First, the whole genome was partitioned into 100-bp long bins, and the average ChIP signal for each bin was calculated. Initially, the ChIP signal values from five bins were subjected to double *t*-test. If the *p*-values from the double *t*-test were less than 0.01, then these 5 bins were used as a seed window to slide across the genome with a 1 bin step size. Double *t*-test was performed for each step until a *p*-value of >0.01 was encountered or the number of bins reached fifty. Second, only the regions that were identified as differentially enriched based on statistical analysis and that possessed a minimum of a 2-fold change in the ChIP signal between the two samples were kept. The differential regions that were separated by gaps of size <1kb were further merged. Finally, we applied an algorithm previously described by Adli et al to evaluate signal enrichment in these regions with small adjustments [38]. Briefly, we first established the background by calculating the mean signal value of an extended region of 10kb covering the differential region identified above. Then, for each 100-bp window sliding across each differential region in 1bp steps, we calculated a Poisson *p*-value by comparing the window value to the background. For H3K4me3 and H3K9ac, windows with Poisson *p*-value <10E−5 were identified as enriched, and for H3K9me3 and H3K27me3, windows with Poisson *p*-value <10E−3 were identified as enriched. The differential regions that did not contain enriched regions in both of the samples were filtered out.

### Pathway Enrichment Analysis

We collated a set of 25,489 RefSeq genes (US National Center for Biotechnology Information; April 18, 2010 update). Pathway enrichment analysis was performed using the KEGG (Kyoto Encyclopedia of Genes and Genomes) web server (http://bioinfo.vanderbilt.edu/webgestalt/) and the DAVID (Database for Annotation, Visualization and Integrated Discovery) web server (http://david.abcc.ncifcrf.gov/).

### Quantitative Real-time PCR and Statistical Analysis

Total RNA isolation and reverse transcription were performed as previously described [35]. Primers for real-time quantitative PCR (RT-qPCR) were designed using Primer 5 software and listed in Table S2. Real-time qPCR analysis was performed on ABI Prism 7700 (Applied Biosystems, Tokyo, Japan) using SYBR Green real-time PCR master mix (Toyobo Co., Japan). Each measurement was repeated at least in triplicate and normalized to the corresponding GAPDH content values. Relative expression levels of objective mRNAs were calculated using the ΔΔCt method. For gene-specific ChIP analyses, RT-qPCR was used to determine the enrichment of immunoprecipitated DNA relative to the input DNA using gene-specific primer sets. Primers were designed based on differential histone modification regions (DHMRs) in promoters (as indicated in Figure 3) and listed in Table S3. The assays were carried out in three replicates and relative histone modification fold change was calculated for each sample using the ΔΔCt method. All data are presented as the mean values ± S.E. Comparisons were made using the Student’s *t-*test and a two-sided *p-*value <0.05 was considered to indicate statistical significance.

### Data Deposition

The ChIP-seq data generated for this work have been deposited in the NCBI Gene Expression Omnibus (GEO) (http://www.ncbi.nlm.nih.gov/geo/) and are accessible through GEO Series accession number GSE41479.

## Supporting Information

Figure S1 **Sorafenib abolished the migratory capacity of A549 alveolar epithelial cells.** A549 cells cultured with TGF-β1 (5 ng/ml) in the absence or presence of sorafenib (5 µM) were subjected to *in vitro* scratch assay with images captured at 0 h and 24 h after incubation using a phase-contrast microscope. The edge of the scratch is marked by imaginary dashed lines.
(TIF)

Figure S2 **Overview of histone modifications in the genic and promoter regions.** (A) Distribution of histone modification signals in genic regions for H3K4me3, H3K9ac, H3K27me3 and H3K9me3. The mRNA region of each gene (from TSS to TTS) was divided into 100 portions, and the up-5k and down-5k region of each gene was divided into 25 portions, respectively. The X-axis indicates the relative distance to the TSS, the Y-axis indicates the intensity of each histone mark and the curve represents the mean signal of all genes in this region. (B) Box plots show the intensities of histone modification signals in the promoter regions of all genes. The promoter is defined as a region crossing upstream 2000 base pairs (bp) and downstream 2000 bp of the transcriptional start site (TSS). The black line in the box represents the mid-value of the signals for all of the promoters. Z-test was used to identify significant differences between control and TGF-β1-treated cells or between control and TGF-β1+sorafenib-treated cells. Significance is indicated by ** based on a threshold of *p*-value <1E-5.
(TIF)

Figure S3 **Treatment with sorafenib counteracts TGF-β1-induced EMT in human lung adenocarcinoma PC9 cells.** (A) PC9 cells were treated with TGF-β1 (5 ng/mL) and/or sorafenib (5 µM) for 48 h, lysed and immunoblotted using antibodies as indicated. (B) H3K4me3 modification and RNA expression changes in PC9 cells. The data are representative of three similar experiments and are displayed as the mean ± SE. **, *p*<0.01 as evaluated using the Student’s *t*-test.
(TIF)

Figure S4 **Sorafenib regulates the expression levels of DNMTs.** After treatment with TGF-β1 (5 ng/ml) in the absence or presence of sorafenib (5 µM) for 24 h and 48 h, A549 cells were subjected to real-time qRT-PCR analysis to determine the effect of sorafenib on the expression levels of *DNMT1*, *DNMT3A* and *DNMT3B*. Normal A549 cells without any treatment (at 0 h) were taken as the control. The quantitative ratios are normalized to the expression of GAPDH. The data are representative of six similar experiments and are displayed as the mean ± SE. **, *p*<0.01 as evaluated using the Student’s *t*-test.
(TIF)

Table S1 **Summary of uniquely mapped reads per histone marker.**
(XLS)

Table S2 **Optimized primers used for real-time qPCR.**
(XLS)

Table S3 **Primers used for H3K4me3 ChIP-qPCR.**
(XLS)

Dataset S1 **DHMGs reciprocally regulated by sorafenib during EMT and KEGG Pathway Enrichment Analysis.** The file contains the lists of DHMGs including H3K4me3, H3K9ac, H3K9me3 and H3K27me3 that are reciprocally regulated by sorafenib during EMT, and KEGG Pathway Enrichment Analysis of these DHMGs.
(XLS)

Dataset S2 **DHMGs during EMT and KEGG Pathway Enrichment Analysis.** The file contains the lists of DHMGs during EMT including H3K4me3, H3K9ac, H3K9me3 and H3K27me3, and KEGG Pathway Enrichment Analysis of these DHMGs.
(XLS)

Dataset S3 **DAVID Pathway Enrichment Analysis of DHMGs reciprocally regulated by sorafenib during EMT.** The file contains DAVID Pathway Enrichment Analysis of DHMGs reciprocally regulated by sorafenib during EMT.
(XLS)
