# Supplementary figures and images for: Sorafenib Inhibits Epithelial-Mesenchymal Transition through an Epigenetic-Based Mechanism in Human Lung Epithelial Cells
Source: PLoS One. 2013 May 31;8(5):e64954. doi: 10.1371/journal.pone.0064954 (PMC3669213; doi:10.1371/journal.pone.0064954)

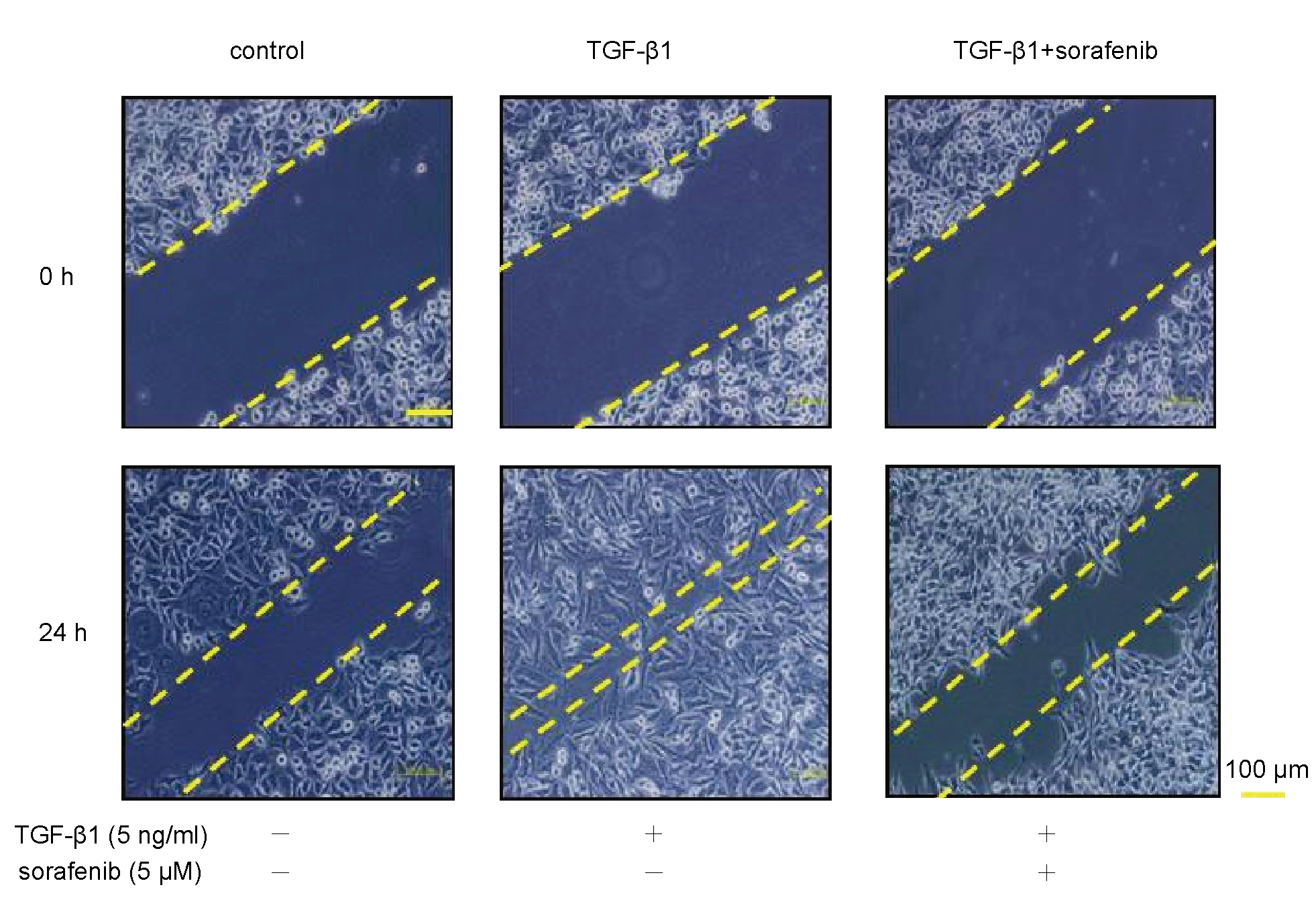

Supplement: Figure S1 — Sorafenib abolished the migratory capacity of A549 alveolar epithelial cells. A549 cells cultured with TGF-β1 (5 ng/ml) in the absence or presence of sorafenib (5 µM) were subjected to in vitro scratch assay with images captured at 0 h and 24 h after incubation using a phase-contrast microscope. The edge of the scratch is marked by imaginary dashed lines. (TIF) [file pone.0064954.s001.tif]

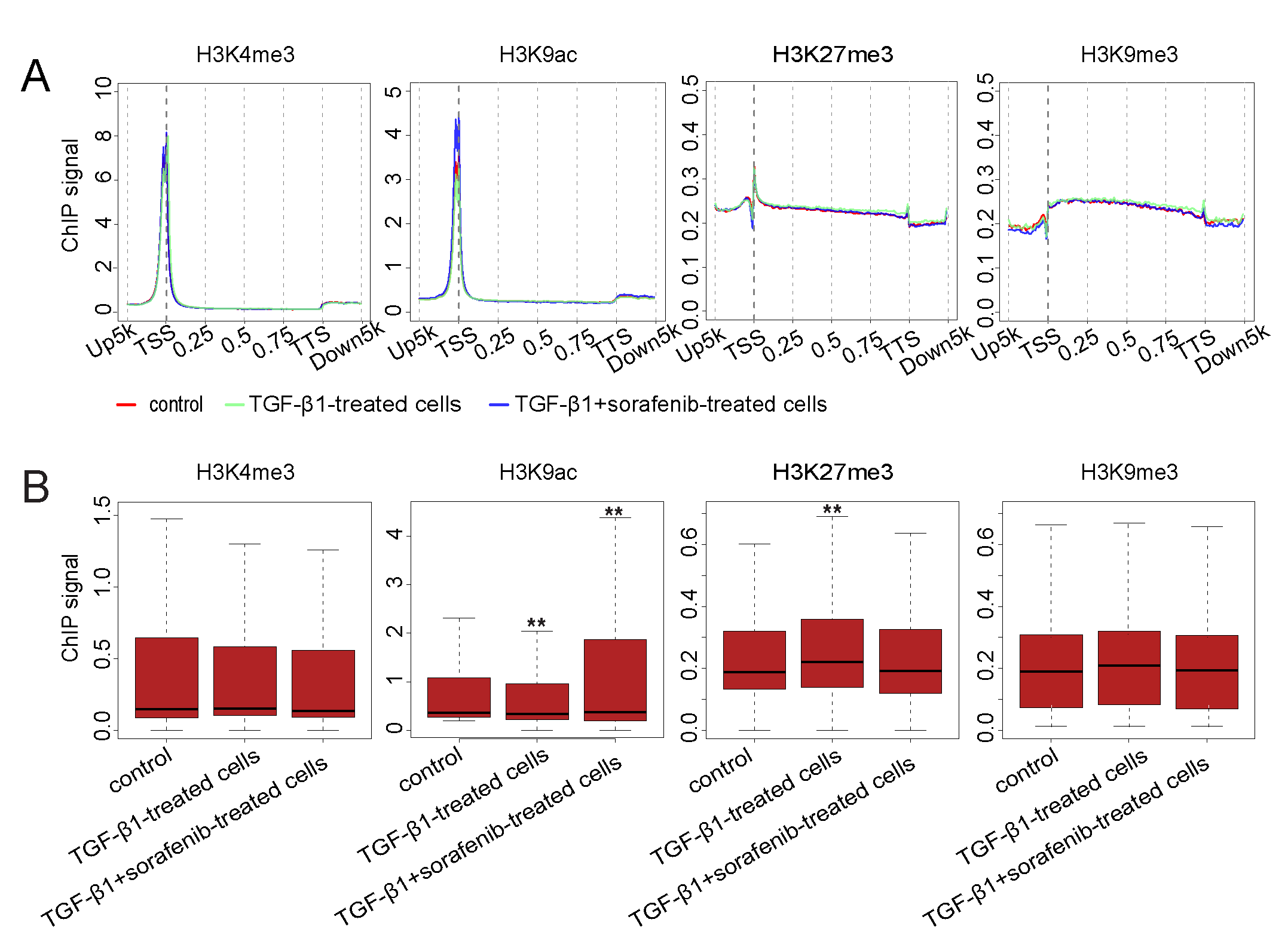

Supplement: Figure S2 — Overview of histone modifications in the genic and promoter regions. (A) Distribution of histone modification signals in genic regions for H3K4me3, H3K9ac, H3K27me3 and H3K9me3. The mRNA region of each gene (from TSS to TTS) was divided into 100 portions, and the up-5k and down-5k region of each gene was divided into 25 portions, respectively. The X-axis indicates the relative distance to the TSS, the Y-axis indicates the intensity of each histone mark and the curve represents the mean signal of all genes in this region. (B) Box plots show the intensities of histone modification signals in the promoter regions of all genes. The promoter is defined as a region crossing upstream 2000 base pairs (bp) and downstream 2000 bp of the transcriptional start site (TSS). The black line in the box represents the mid-value of the signals for all of the promoters. Z-test was used to identify significant differences between control and TGF-β1-treated cells or between control and TGF-β1+sorafenib-treated cells. Significance is indicated by ** based on a threshold of p-value <1E-5. (TIF) [file pone.0064954.s002.tif]

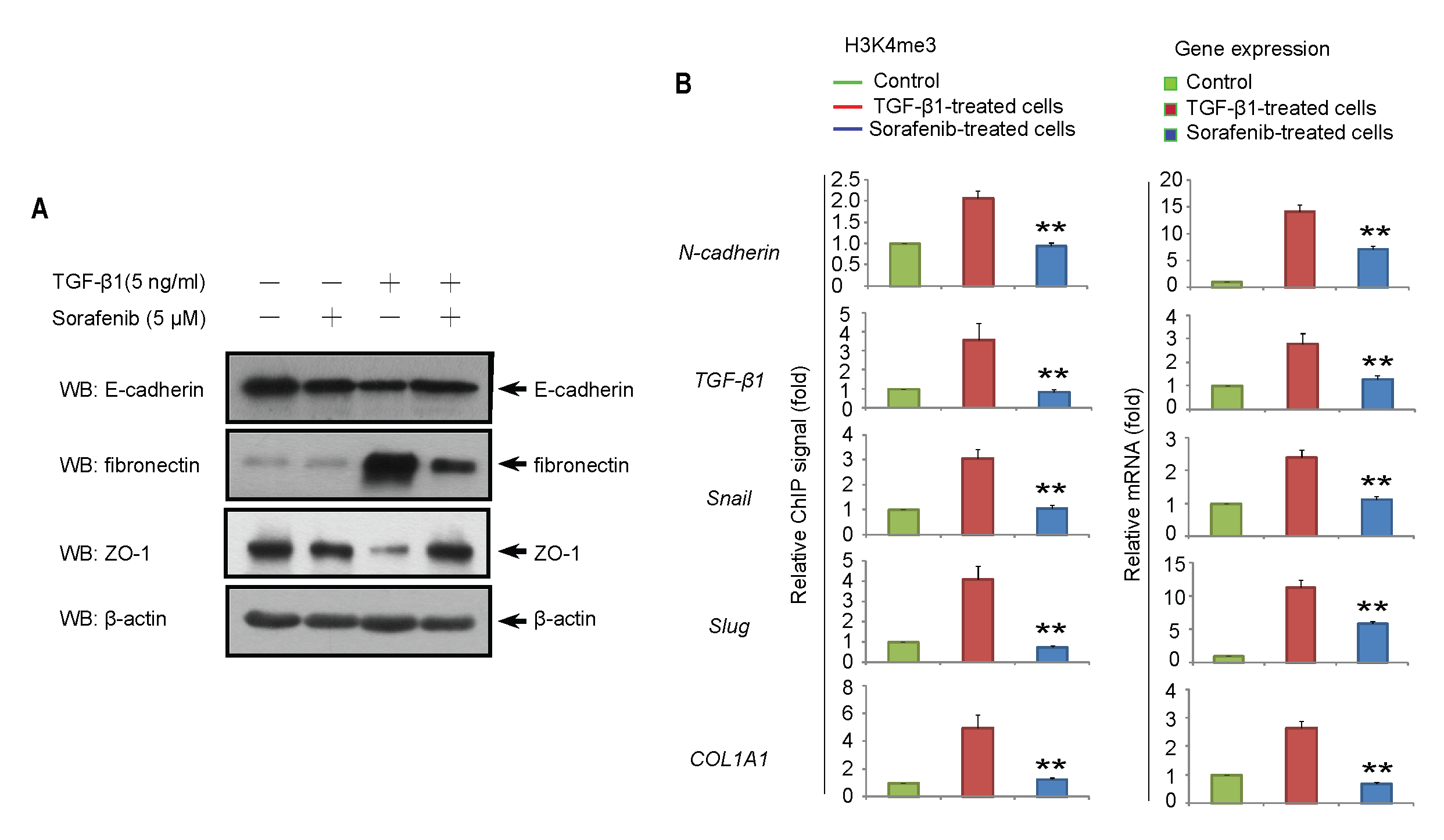

Supplement: Figure S3 — Treatment with sorafenib counteracts TGF-β1-induced EMT in human lung adenocarcinoma PC9 cells. (A) PC9 cells were treated with TGF-β1 (5 ng/mL) and/or sorafenib (5 µM) for 48 h, lysed and immunoblotted using antibodies as indicated. (B) H3K4me3 modification and RNA expression changes in PC9 cells. The data are representative of three similar experiments and are displayed as the mean ± SE. **, p<0.01 as evaluated using the Student’s t-test. (TIF) [file pone.0064954.s003.tif]

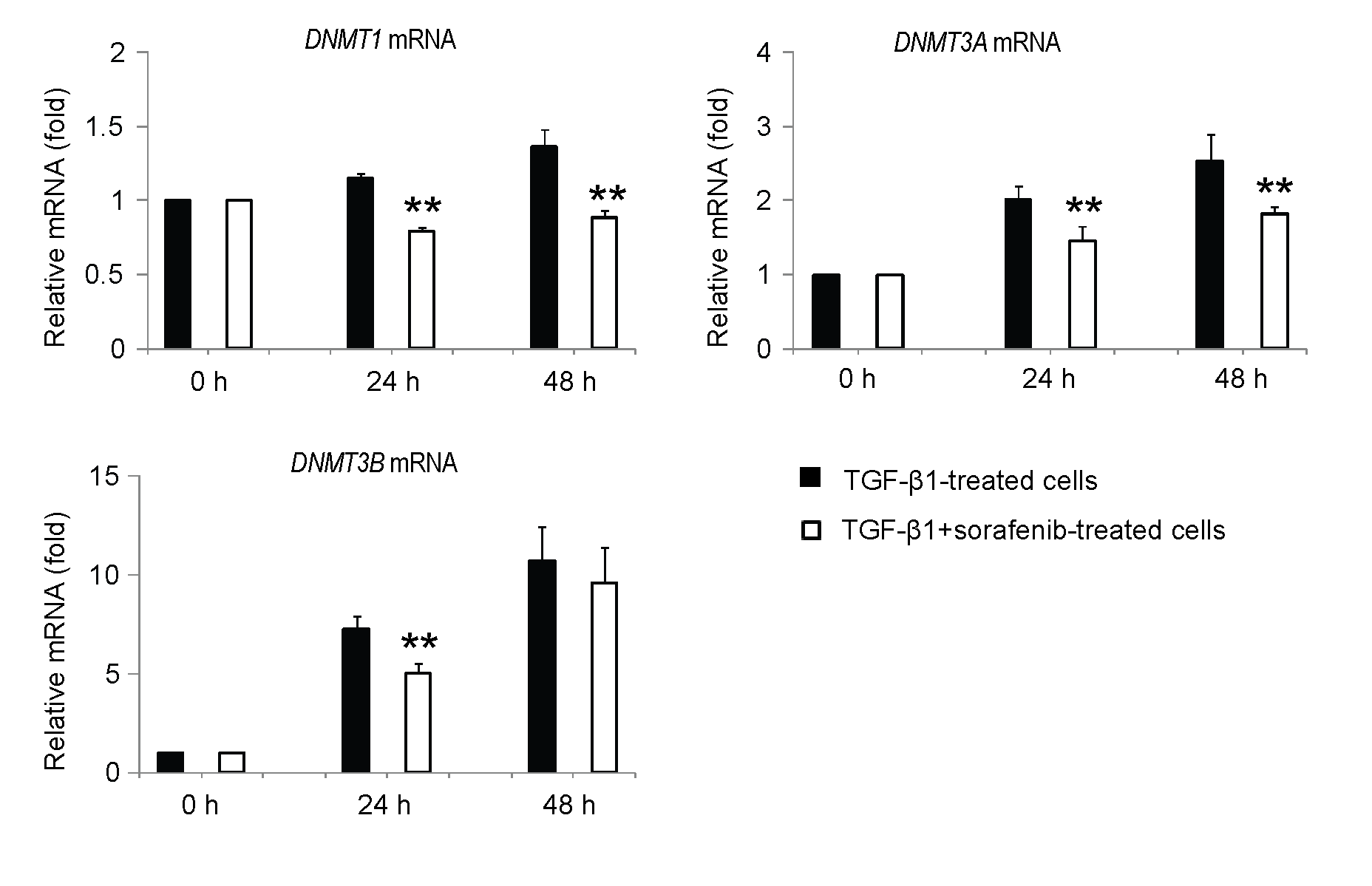

Supplement: Figure S4 — Sorafenib regulates the expression levels of DNMTs. After treatment with TGF-β1 (5 ng/ml) in the absence or presence of sorafenib (5 µM) for 24 h and 48 h, A549 cells were subjected to real-time qRT-PCR analysis to determine the effect of sorafenib on the expression levels of DNMT1, DNMT3A and DNMT3B. Normal A549 cells without any treatment (at 0 h) were taken as the control. The quantitative ratios are normalized to the expression of GAPDH. The data are representative of six similar experiments and are displayed as the mean ± SE. **, p<0.01 as evaluated using the Student’s t-test. (TIF) [file pone.0064954.s004.tif]
